# Efficacy and safety of transarterial chemoembolization combined with targeted therapy and immunotherapy versus with targeted monotherapy in unresectable hepatocellular carcinoma: A systematic review and meta-analysis

**DOI:** 10.1097/MD.0000000000038037

**Published:** 2024-05-03

**Authors:** Jingwen Feng, Yi Zhao, Lin Zhai, Jingxu Zhou

**Affiliations:** a The First Clinical School of Guangzhou University of Chinese Medicine, Guangzhou, China; b Yueyang Hospital of Integrated Traditional Chinese and Western Medicine, Shanghai University of Traditional Chinese Medicine, Shanghai, China; c The First Affiliated Hospital of Guangzhou University of Chinese Medicine Cancer Center, Guangzhou, China.

**Keywords:** efficacy, immunotherapy, meta-analysis, safety, targeted therapy, transarterial chemoembolization, unresectable hepatocellular carcinoma

## Abstract

**Background and objective:**

The application of transarterial chemoembolization (TACE) in combination with targeted therapy and immunotherapy (TACE-T-I) for unresectable hepatocellular carcinoma (HCC) has gained increasing attention. However, there are variations in the efficacy and safety outcomes between TACE-T-I versus TACE combined with targeted drugs (TACE-T). This study aims to systematically evaluate the efficacy and safety of TACE-T-I versus TACE-T in unresectable HCC.

**Methods:**

PubMed, Embase, Cochrane Library, and Web of Science databases were searched from inception to August 21, 2023, for comparative studies on TACE-T-I versus TACE-T for unresectable HCC. Outcome measures included overall survival (OS), progression-free survival (PFS), objective response rate (ORR), disease control rate (DCR) and the incidence of treatment-related adverse events (TRAEs). OS was the primary outcome of this study. Weighted mean difference (WMD) or hazard ratio (HR) was used as the pooled statistic for OS and PFS. Relative risk (RR) was employed as the pooled statistic for ORR, DCR and the incidence of TRAEs. And 95% confidence intervals (CIs) were calculated for all effect measures. Data analysis was conducted using Stata 14.0 software.

**Results:**

The meta-analysis included 14 studies with 2144 patients. The pooled results showed that compared with patients in the TACE-T group, patients in the TACE-T-I group had higher ORR (RR = 1.61; 95%CI: 1.38–1.89) and DCR (RR = 1.17; 95%CI: 1.09–1.26). Patients in the TACE-T-I group experienced prolonged PFS (WMD = 3.08; 95%CI: 2.63–3.53) and OS (WMD = 5.76; 95%CI: 4.68–6.84). And the risk of disease progression (HR = 0.45; 95%CI: 0.37–0.55) and death (HR = 0.43; 95%CI: 0.38–0.49) was lower in the TACE-T-I group. Common TRAEs included fever, pain, abdominal pain, nausea, vomiting, elevated ALT, elevated AST, hypertension, hand-foot syndrome, proteinuria, and diarrhea. The incidence and severity of TRAEs in the TACE-T-I group were similar to those in the TACE-T group, with no significant differences (*P* > .05).

**Conclusion:**

Current evidence suggests that, on the basis of TACE combined with targeted therapy, the addition of immunotherapy provides better clinical efficacy and survival benefits for unresectable HCC patients, with good tolerability.

## 1. Introduction

Hepatocellular carcinoma (HCC) ranks as one of the most common malignancies, with the sixth highest incidence and the third highest mortality worldwide, resulting in approximately 906,000 new diagnoses annually.^[1]^ More than half of HCC cases occur in developing countries, particularly in Southeast Asian nations.^[2]^ HCC accounts for 75% to 85% of primary liver cancers, being the predominant pathological type. Unfortunately, a significant proportion of patients are diagnosed at an advanced-stage, precluding curative surgical resection, leading to poor prognosis.^[3]^ Thus, improving the prognosis and extending survival represent the primary goals and challenges in the current management of patients with intermediate and advanced HCC.

Transarterial chemoembolization (TACE) is presently recommended as the standard first-line treatment for unresectable intermediate-stage HCC by multiple guidelines.^[3]^ It is also commonly employed as a palliative treatment for advanced-stage HCC. Nearly 50% of patients with BCLC (Barcelona Clinic Liver Cancer)-C stage chose TACE as the first-line treatment.^[4]^ TACE can be classified into conventional TACE (cTACE) and drug-eluting beads TACE (DEB-TACE) based on the type of embolic agent used.^[3]^ However, the efficacy of TACE is often suboptimal for advanced-stage HCC, characterized by extensive tumor burden, vascular invasion, liver dysfunction, and the potential for extrahepatic metastases.^[5]^ Additionally, post-TACE residual tumor foci and the local hypoxic microenvironment upregulate the expression of vascular endothelial growth factor and fibroblast growth factor, promoting local recurrence and distant metastasis, thereby limiting the long-term effectiveness of TACE.^[6]^ Combining TACE with multikinase inhibitors possessing anti-proliferative and anti-angiogenic effects represents a potential strategy to overcome these limitations.^[7]^ The phase II clinical trial (TACTICS trial), led by Professor Kudo and colleagues in Japan, demonstrated that the combination of TACE and sorafenib extended the progression-free survival (PFS) of patients to 25.2 months, compared to 13.5 months in the TACE monotherapy group.^[8]^ Similarly, Chinese scholars reported that the combination of TACE and apatinib prolonged PFS in patients with intermediate and advanced-stage HCC.^[9]^ Therefore, the combination of TACE with molecular-targeted drugs presents a novel therapeutic approach for intermediate and advanced-stage HCC. Recently, immunotherapy has shown remarkable efficacy in liver cancer. In 2020, the combination of atezolizumab (Atezo) and bevacizumab (Bev) demonstrated superior treatment outcomes compared to sorafenib alone.^[5]^ Consequently, the combination of targeted therapy and immunotherapy has become the standard systemic treatment for advanced HCC, endorsed by multiple guidelines as a combined treatment approach for intermediate-stage HCC.^[10,11]^ Furthermore, the CHANCE2211 study demonstrated that TACE plus camrelizumab and apatinib showed significantly better OS, PFS, and ORR versus TACE monotherapy for advanced HCC.^[12]^ The application of TACE in combination with targeted therapy and immunotherapy (TACE-T-I) gains increasing attention for unresectable HCC,^[6]^ and the triple combination is becoming one of the clinical treatment choices for more and more advanced HCC patients.

To date, TACE-based combination therapies have been regarded as fundamental choice for unresectable HCC. And the triple combination therapy showed promising efficacy and safety in patients with advanced HCC.^[13]^ However, the differences in efficacy and safety between the dual combination (TACE-T) and the triple combination (TACE-T-I) have not yet been demonstrated. The results of these studies^[14–18]^ were inconsistent. Therefore, this paper aims to conduct a systematic review and meta-analysis, consolidating relevant research evidence to evaluate whether TACE-T-I provides significant clinical benefits compared to TACE-T for patients with unresectable HCC. The goal is to provide theoretical foundations for the clinical treatment strategies of intermediate and advanced-stage HCC.

## 2. Materials and methods

### 2.1. Search strategy

Computerized searches were conducted in PubMed, Embase, Cochrane Library, and Web of Science to collect comparative studies on TACE-T-I versus TACE-T for unresectable HCC. The search was conducted from the inception of the databases until August 21, 2023. Additionally, reference lists of included studies were manually searched, and relevant reviews were screened to identify additional studies suitable for this meta-analysis. We established search strategies that combined database-specific subject headings (such as MeSH terms) and free text terms. The search terms used to define the therapy included (“transcatheter arterial chemoembolization” OR “tace” OR “transarterial chemoembolization”) AND (“target” OR “targeted” OR “sorafenib” OR “lenvatinib” OR “regorafenib” OR “apatinib” OR “bevacizumab”) AND (“immunotherapy” OR “immunological therapy” OR “immune checkpoint inhibitors” OR “pd 1 inhibitor” OR “atezolizumab” OR “pembrolizumab” OR “nivolumab” OR “camrelizumab” OR “sintilimab” OR “toripalimab”). The terms used to define the disease included “liver neoplasms” OR “liver cancer” OR “hepatocellular carcinoma.” The terms of the same group were combined with “OR” and different groups were combined with “AND,” with adjustments made based on the characteristics of each database. Specific search strategies for each database are outlined in Tables S1 to S4, Supplemental Digital Content 1–4, http://links.lww.com/MD/M399, http://links.lww.com/MD/M400, http://links.lww.com/MD/M401, http://links.lww.com/MD/M402.

### 2.2. Study selection

#### 2.2.1. Inclusion criteria

The inclusion criteria were as follows: Studies focused on patients with unresectable HCC diagnosed clinically or histopathologically; The TACE-T-I group received TACE combined with targeted therapy (targeted medicine) and immunotherapy, while the TACE-T group received TACE with targeted therapy alone; Study designs include randomized controlled trials (RCTs) and non-RCTs; Outcome measurements included overall survival (OS), progression-free survival (PFS), objective response rate (ORR), disease control rate (DCR), treatment-related adverse events (TRAEs).

#### 2.2.2. Exclusion criteria

The studies were excluded if they met the following criteria: Reviews, conference abstracts, and comments for which data were unavailable. Studies using different targeted drugs in the 2 groups. For duplicate publications or the same data in multiple articles, the most complete and recent literature was retained, and others were excluded.

### 2.3. Data extraction

Two researchers independently screened the literature, extracted data, and then cross-checked their results. Disagreements were resolved through discussion or consultation with a third researcher. Following the inclusion and exclusion criteria, researchers independently completed the literature selection. After determining the included studies, data extraction was independently conducted using a pre-designed standardized form. After 2 researchers completed the data extraction, the extraction tables were exchanged for verification. Any discrepancies were resolved through discussion or negotiation. For each eligible study, the following information was extracted: first author, publication year, study location, study design, baseline characteristics of study participants, sample size, intervention measures, follow-up duration, and study outcomes.

### 2.4. Quality assessment

The ROBINS-I tool was used to assess the risk of bias in non-randomized clinical trials, while the Cochrane Collaboration tool was employed for quality evaluation of RCTs.^[19,20]^ Two researchers independently performed and cross-checked the assessments. Discrepancies were resolved through discussion or consultation with a third party.

### 2.5. Statistical analysis

Stata 14.0 software was utilized for data analysis. Weighted mean difference (WMD) was used as the pooled statistic for OS and PFS, while hazard ratio (HR) was used for comparing the difference in the risk of disease progression and death. Relative risk (RR) was employed as the pooled statistic for other study outcomes, including ORR, DCR, and the incidence of TRAEs. Meanwhile, 95% confidence intervals (CIs) were calculated for all effect measures.

A *P* value <0.05 indicated statistical significance. Considering the significant clinical and methodological heterogeneity among included studies, a random-effects model was applied for meta-analysis.^[21]^ Heterogeneity between studies was evaluated by Cochran Q-test and inconsistency index (I^2^).^[22]^ Significant statistical heterogeneity was considered if *P* < .1 and heterogeneity I^2^ > 50%; otherwise, heterogeneity was not significant. Subgroup analyses were conducted based on different study types. Sensitivity analysis was performed using the leave-one-out method to assess the stability of the pooled results. Funnel plots, Egger test, and the trim-and-fill method were employed to detect publication bias.

## 3. Results

### 3.1. Literature selection

**Figure 1.**
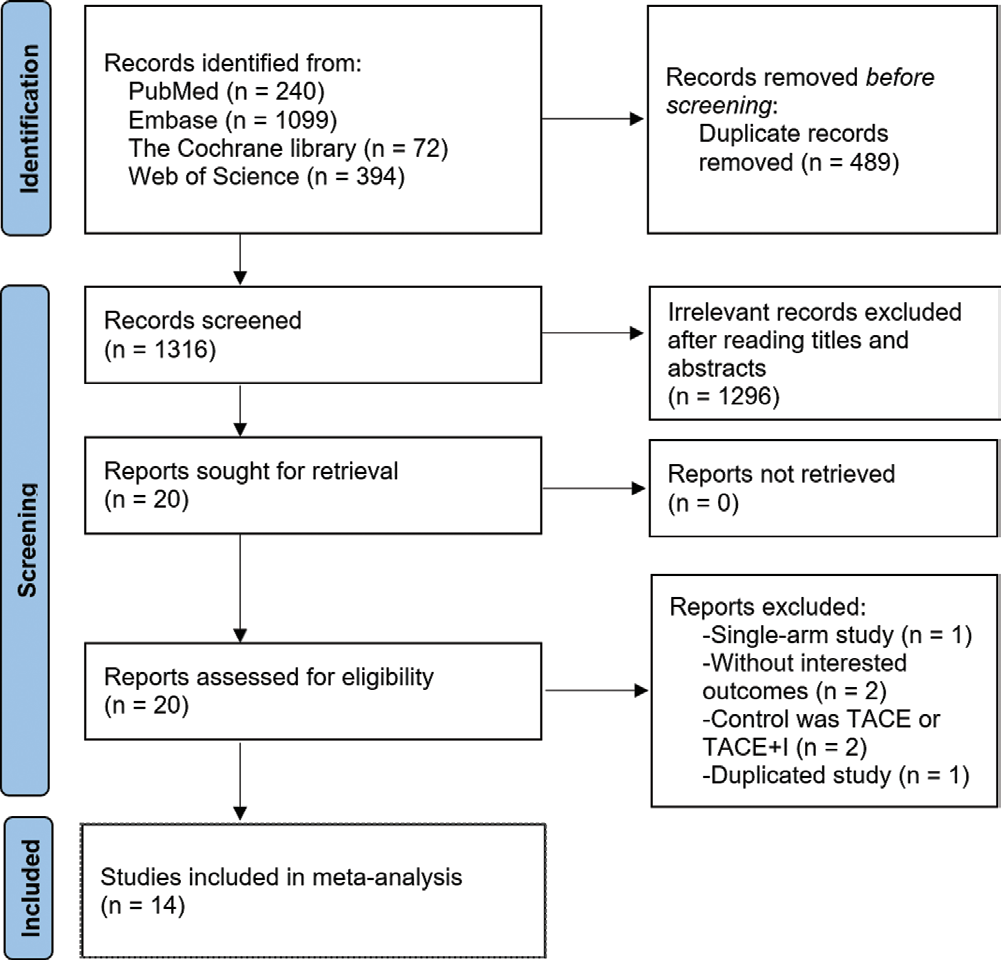
PRISMA flow diagram showing screening and selection process.

As shown in Figure 1, a total of 1805 articles were identified through database searches, with 489 duplicates removed. After the initial screening, 1296 articles were excluded, followed by the exclusion of an additional 6 articles during the second screening. No additional studies were identified through reference tracing. Ultimately, 14 articles were included in the analysis.^[14–18,23–31]^

### 3.2. Characteristics of included studies

The included articles were published between 2021 and 2023, all originating from China. The majority of articles (n = 14) were retrospective cohort studies,^[14–18,23,24,26–31]^ and one was a prospective real-world study.^[24]^ There were 2144 patients, with 1013 in the TACE-T-I group and 1131 in the TACE-T group. The basic characteristics of the included studies are presented in Table 1.

**Table 1:** Characteristics of the included studies in this meta-analysis.

Study	Country	Design	ECOG PS	Child-Pugh class	BCLC stage	Type of TACE	TACE-T-I/ TACE-T		Targeted medicine	Immunotherapy drug
							Cases	Age, yr		
Cai et al^[10]^	China	RCS	≤1	A/B	C	cTACE or DEB-TACE	41/40	51.9/54.6	Lenvatinib, 12 mg (bodyweight ≥ 60 kg) or 8 mg (bodyweight < 60 kg), orally once a d	Sintilimab, Tislelizumab or camrelizumab, 200 mg intravenous injection, every 3 wk
Chen et al^[11]^	China	RCS	≤1	A	B/C	DEB-TACE	70/72	58/57	Lenvatinib, 8 mg (regardless of bodyweight), orally once a d	Pembrolizumab, 200 mg intravenous injection, every 3 wk
Duan et al^[19]^	China	RCS	≤1	A/B	B/C	cTACE or DEB-TACE	483/477	52.6/52.9	Apatinib, given orally at 250 mg/d	Camrelizumab, 200 mg intravenously every 3 wk
Jiang et al^[12]^	China	RCS	≤1	A/B	B/C	cTACE or DEB-TACE	42/45	61.7/61.2	Sorafenib 0.4 g orally twice daily, lenvatinib 12 mg (≥60 kg) or 8 mg (<60 kg) orally once daily, apatinib 750 mg orally once daily, regorafenib 160 mg orally once daily, and bevacizumab 15 mg/kg intravenously every 3 wk	Camrelizumab, Sintilimab, Pembrolizumab, Tislelizumab (200 mg), and Atezolizumab (1200 mg), intravenously every 3 wk
Liu et al^[20]^	China	RCS	≤1	A/B	B/C	cTACE	37/39	NR	Apatinib, given orally at 250 mg/d, for 4 wk	Camrelizumab, 200 mg intravenously, once every 3 wk
Qu et al^[21]^	China	RWS	NR	A/B	B/C	DEB-TACE	30/21	55.5/50.0	Lenvatinib, 8 mg (regardless of bodyweight), orally once a d	Anti-PD-1 antibody, 240 mg intravenously every 3 wk
Sun et al^[13]^	China	RCS	≤1	A/B	B/C	cTACE	31/52	54.8/51.8	Lenvatinib, 12 mg (bodyweight ≥ 60 kg) or 8 mg (bodyweight < 60 kg), orally once a d	Camrelizumab, 200 mg intravenously every 3 wk
Wang et al^[22]^	China	RCS	≤1	A/B	NR	cTACE	54/45	57.0/60.8	Lenvatinib, 8 mg (<60 kg) or 12 mg (≥60 kg) once daily based on body weight	Sintilimab, 200 mg; Toripalimab, 240 mg; Camrelizumab, 200 mg, intravenously every 3 wk
Wang et al^[23]^	China	RCS	≤1	A/B	A/B/C	cTACE	45/20	54/62	Lenvatinib, 12 mg (bodyweight ≥ 60 kg) or 8 mg (bodyweight < 60 kg), orally once a d	Camrelizumab, Sintilimab, Pembrolizumab and Tislelizumab (200 mg) & Toripalimab (240 mg) once every 3 wk; Nivolumab (240 mg) once every 2 wk
Xia et al^[24]^	China	RCS	≤1	A/B	C	cTACE	68/148	NR	Apatinib, given orally at 250 mg/d	Sintilimab/Tislelizumab/Pembrolizumab/Camrelizumab, 200 mg intravenously, every 3 wk
Yang et al^[25]^	China	RCS	≤2	A/B	C	cTACE	33/43	54.6/50.9	Sorafenib, 400 mg orally, twice a d	Camrelizumab, Sintilimab, 200 mg intravenously, every 3 wk
Zhao et al^[26]^	China	RCS	≤1	A/B	B/C	DEB-TACE	23/32	52.8/57.4	Sorafenib, 400 mg orally, twice a d; Lenvatinib, 12 mg (bodyweight ≥ 60 kg) or 8 mg (bodyweight < 60 kg), orally once a d	Nivolumab or Toripalimab, 3 mg/kg intravenously, once every 2 wk
Zheng et al^[27]^	China	RCS	≤2	A/B	B/C	cTACE	22/29	NR	Sorafenib, 400 mg orally, twice a d	Nivolumab or Pembrolizumab, 3 mg/kg intravenously, every 3 wk
Zhu et al^[14]^	China	RCS	≤1	A/B	B/C	cTACE	34/68	NR	Apatinib, given orally at 250 mg/d	Camrelizumab, 200 mg intravenously, once every 3 wk

BCLC = Barcelona Clinic Liver Cancer, cTACE = conventional TACE, DEB-TACE = drug-eluting bead TACE, ECOG PS = Eastern Cooperative Oncology Group performance status, NR = not reported, RCS = retrospective cohort study, RWS = real-world study, TACE = Transcatheter arterial chemoembolization.

### 3.3. Quality assessment of included studies

Table 2 displays the results of the quality assessment of the included studies. Some studies exhibited a moderate bias in terms of participant selection, outcome measurement, and selective reporting. Overall, the methodological quality of the included studies was considered moderate.

**Table 2:** Quality assessment of the non-randomized controlled studies (ROBINS-I).

Study	Bias due to confounding	Bias in selection of participants into the study	Bias in classification of interventions	Bias due to deviations from intended interventions	Bias due to missing data	Bias in measurement of outcomes	Bias in selection of the reported result	Overall bias
Cai et al^[10]^	Low	Moderate	Low	Low	Low	Moderate	Low	Moderate
Chen et al^[11]^	Low	Moderate	Low	Low	Low	Moderate	Low	Moderate
Duan et al^[19]^	Low	Low	Low	Low	Low	Moderate	Low	Moderate
Jiang et al^[12]^	Low	Low	Low	Low	Low	Moderate	Low	Moderate
Liu et al^[20]^	Low	Low	Low	Low	Low	Moderate	Low	Moderate
Qu et al^[21]^	Low	Moderate	Low	Low	Low	Low	Low	Moderate
Sun et al^[13]^	Low	Low	Low	Low	Low	Moderate	Moderate	Moderate
Wang et al^[22]^	Low	Moderate	Low	Low	Low	Moderate	Moderate	Moderate
Wang et al^[23]^	Low	Moderate	Low	Low	Low	Moderate	Low	Moderate
Xia et al^[24]^	Low	Low	Low	Low	Low	Moderate	Low	Moderate
Yang et al^[25]^	Low	Moderate	Low	Low	Low	Moderate	Low	Moderate
Zhao et al^[26]^	Low	Low	Low	Low	Low	Moderate	Low	Moderate
Zheng et al^[27]^	Low	Moderate	Low	Low	Low	Moderate	Low	Moderate
Zhu et al^[14]^	Low	Low	Low	Low	Low	Moderate	Low	Moderate

### 3.4. Meta-analysis and subgroup analyses

Given the diversity in TACE types among the included studies, subgroup analyses were conducted based on TACE types (cTACE or DEB-TACE, DEB-TACE, cTACE). Subgroup analysis for the risk of disease progression and death was additionally performed according to whether the HR was adjusted for multiple factors.

#### 3.4.1. Short-term efficacy outcomes

1. ORR

**Figure 2.**
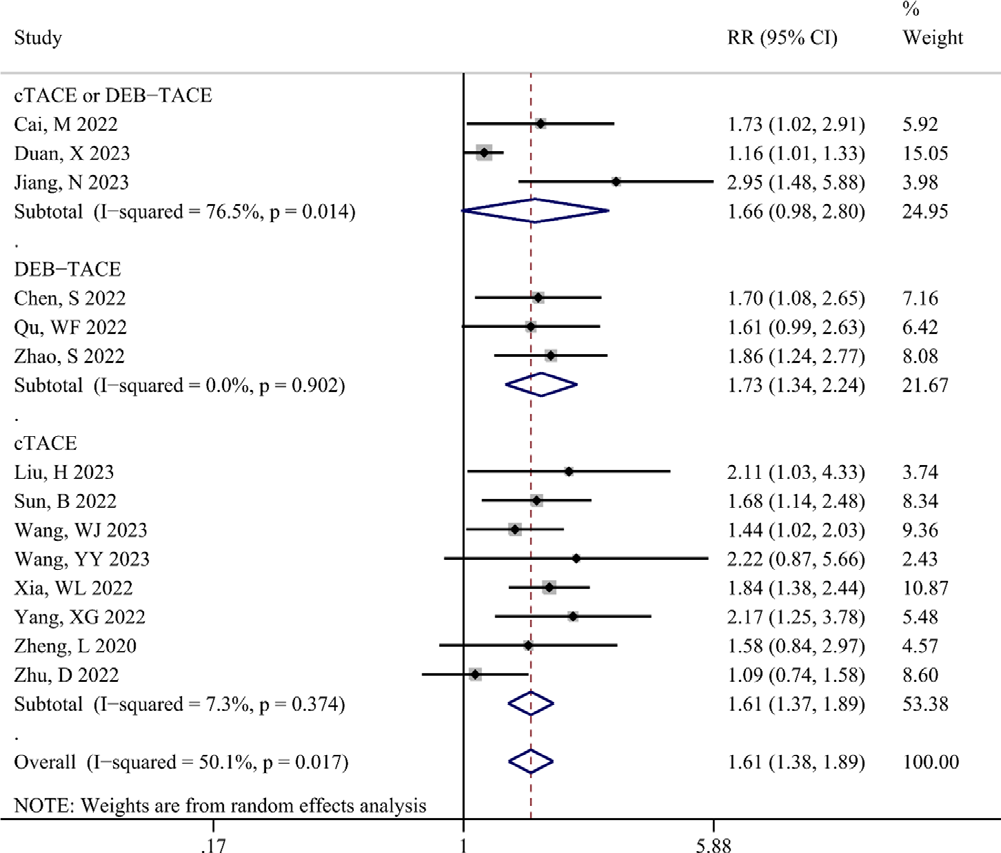
Meta-analysis results of objective response rate (ORR) in TACE-T-I group vs TACE-T group for hepatocellular carcinoma.

The differences in ORR between the TACE-T-I and TACE-T groups are illustrated in Figure 2. The meta-analysis results indicated that the TACE-T-I group had a higher ORR than the TACE-T group, with a statistically significant difference (RR = 1.61; 95% CI: 1.38–1.89; *P* < .001). Subgroup analyses showed no statistically significant differences in ORR between the TACE-T-I and TACE-T groups in the cTACE or DEB-TACE subgroup (RR = 1.66; 95% CI: 0.98–2.80), whereas statistically significant differences were observed in the cTACE subgroup (RR = 1.61; 95% CI: 1.37–1.89) and DEB-TACE subgroup (RR = 1.73; 95% CI: 1.34–2.24). Both the cTACE and DEB-TACE subgroups exhibited minimal statistical heterogeneity (I^2^ < 10%, *P *> .05).

1. DCR

**Figure 3.**
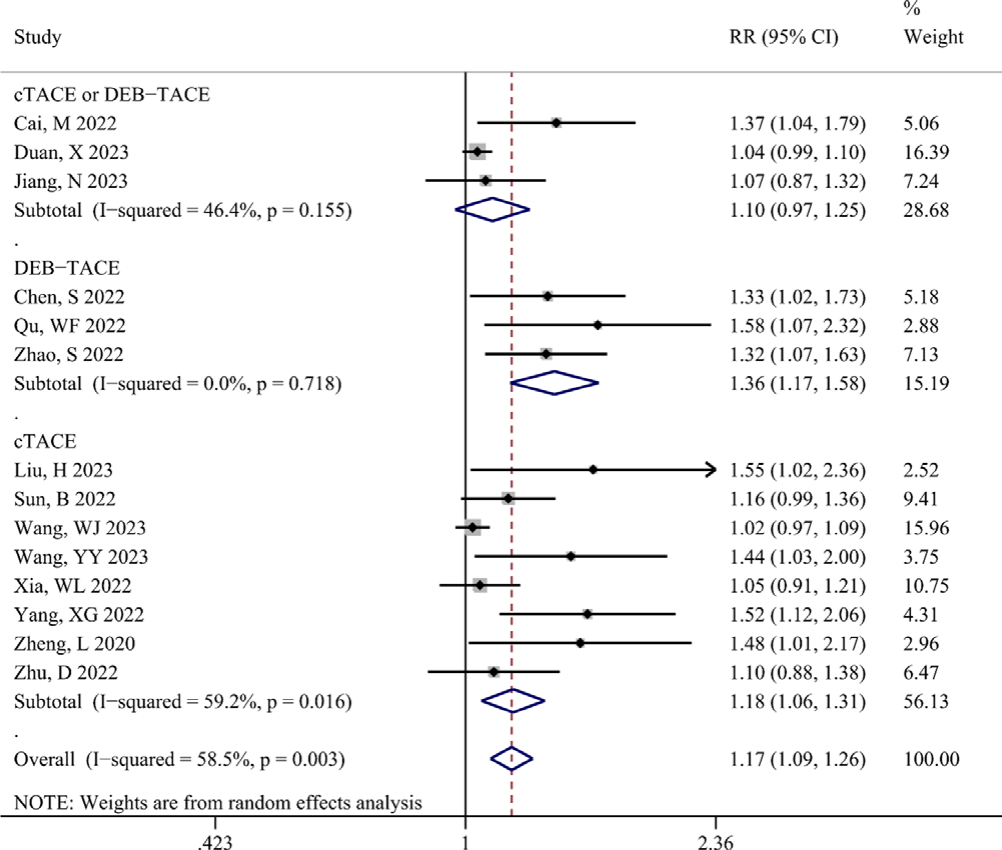
Meta-analysis results of disease control rate (DCR) in TACE-T-I group vs TACE-T group for hepatocellular carcinoma.

The differences in DCR between the TACE-T-I and TACE-T groups are shown in Figure 3. The meta-analysis results indicated that the TACE-T-I group manifested a higher DCR than the TACE-T group (RR = 1.17; 95% CI: 1.09–1.26; *P* < .001). Subgroup analyses showed no statistically significant differences in DCR between the TACE-T-I and TACE-T groups in the cTACE or DEB-TACE subgroup (RR = 1.10; 95% CI: 0.97–1.25), whereas there were statistically significant differences in the cTACE subgroup (RR = 1.18; 95% CI: 1.06–1.31) and DEB-TACE subgroup (RR = 1.36; 95% CI: 1.17–1.58). The cTACE subgroup exhibited significant statistical heterogeneity (I^2^ > 50%, *P* < .05), while the DEB-TACE subgroup showed no heterogeneity (I^2^ = 0.0%, *P* > .05).

#### 3.4.2. Long-term efficacy outcomes

1. PFS

**Figure 4.**
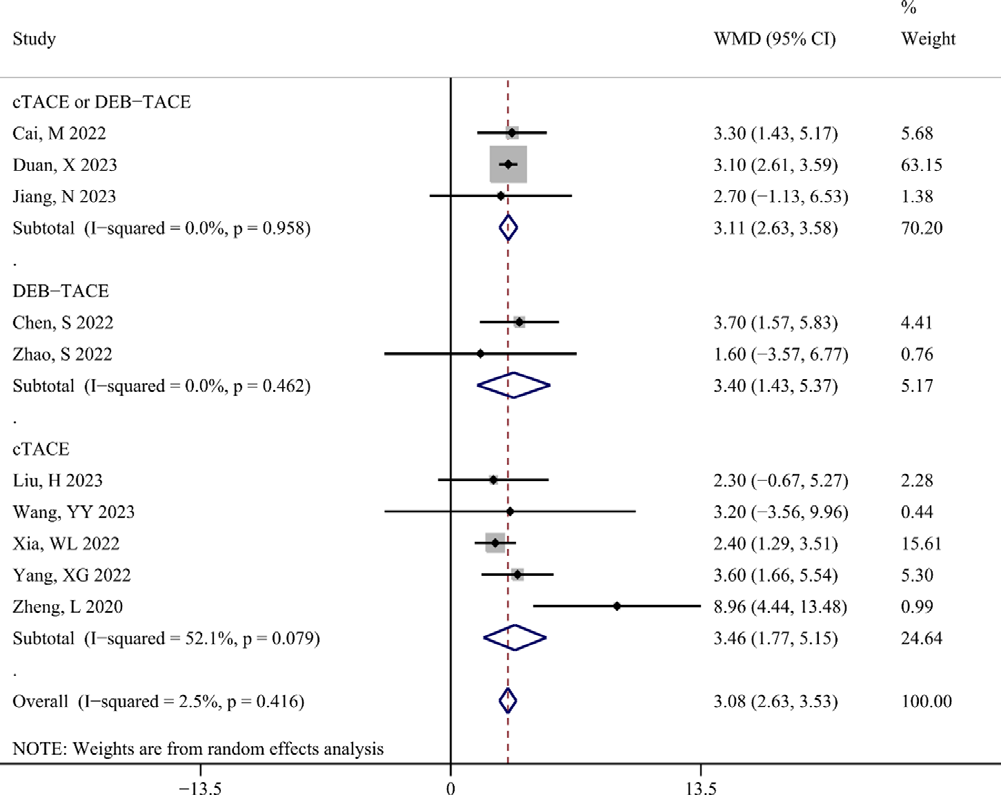
Meta-analysis results of progression-free survival (PFS, in months) in TACE-T-I group vs TACE-T group for hepatocellular carcinoma.

**Figure 5.**
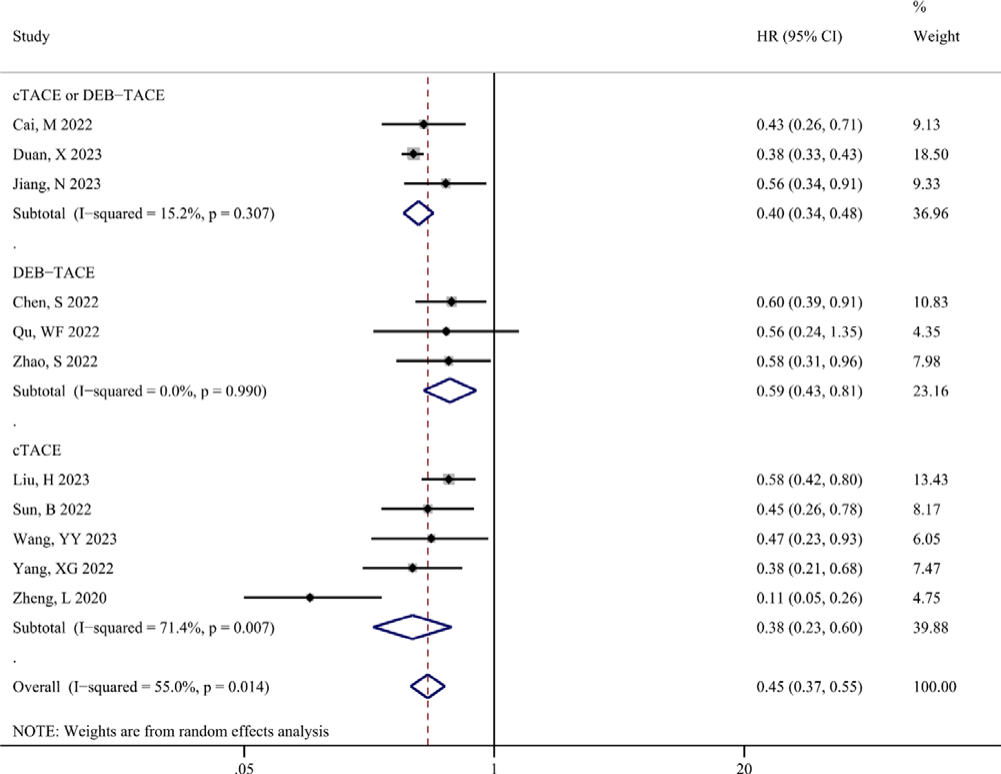
Meta-analysis results of hazard ratio of progression-free survival (PFS) in TACE-T-I group vs TACE-T group for hepatocellular carcinoma.

Figure 4 shows the difference in PFS (in months) between the TACE-T-I and TACE-T groups. The meta-analysis results displayed that the TACE-T-I group had a higher PFS than the TACE-T group (WMD = 3.08; 95% CI: 2.63–3.53; *P* < .001). Figure 5 illustrates the difference in the risk of disease progression between the TACE-T-I and TACE-T groups. A lower risk of disease progression was found in the TACE-T-I group, with a statistically significant difference (HR = 0.45; 95% CI: 0.37–0.55; *P* < .001). Subgroup analyses based on whether the HR was adjusted for multiple factors yielded similar results (Figure S1, Supplemental Digital Content 5, http://links.lww.com/MD/M403). Subgroup analyses by different TACE types also showed consistent results across all subgroups.

1. OS

**Figure 6.**
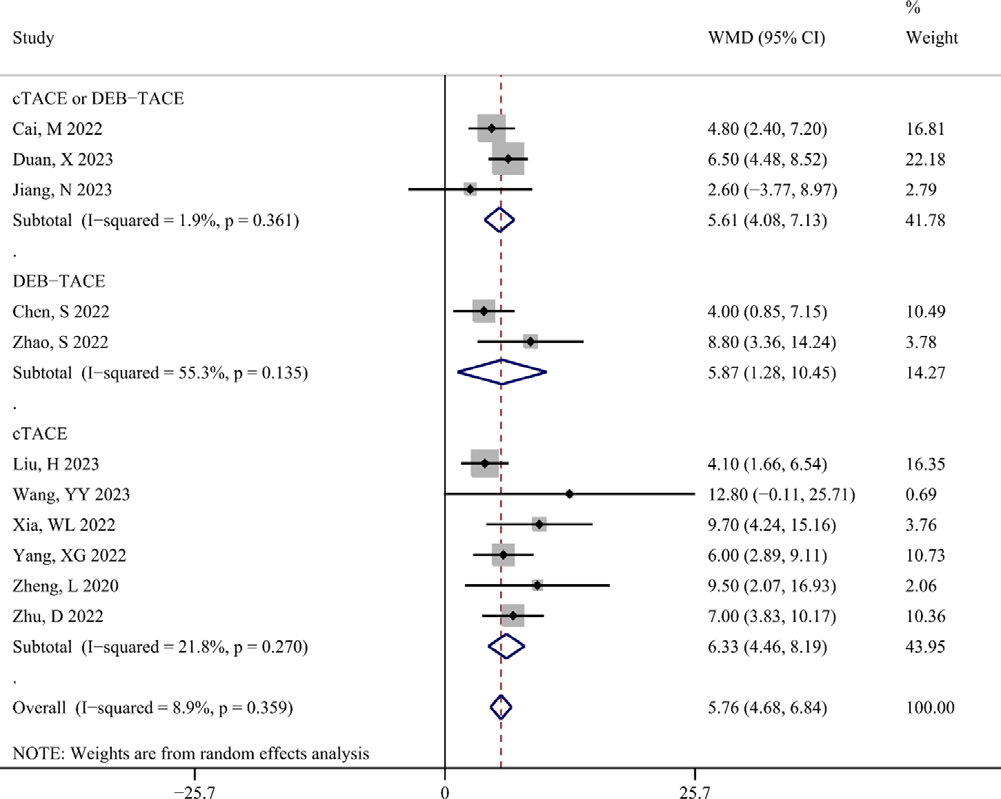
Meta-analysis results of overall survival (OS, in months) in TACE-T-I group vs TACE-T group for hepatocellular carcinoma.

**Figure 7.**
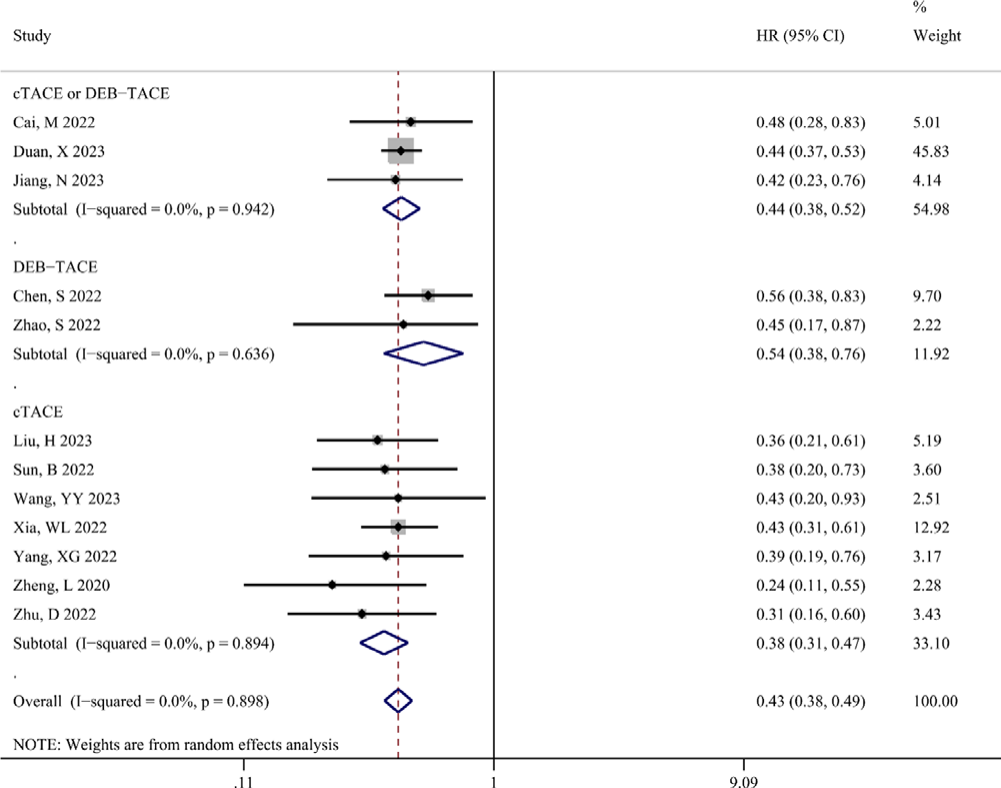
Meta-analysis results of hazard ratio of overall survival (OS) in TACE-T-I group vs TACE-T group for hepatocellular carcinoma.

OS was defined as the primary outcome to assess the impact of TACE combined with targeted therapy and immunotherapy on the survival of patients with unresectable HCC. Figure 6 shows the difference in OS (in months) between the TACE-T-I and TACE-T groups. The meta-analysis results indicated that the TACE-T-I group had a longer OS than the TACE-T group, with a statistically significant difference (WMD = 5.76; 95% CI: 4.68–6.84; *P* < .001). Figure 7 shows the difference in the risk of death for OS between the TACE-T-I and TACE-T groups. The meta-analysis results revealed a lower risk of death for OS in the TACE-T-I group, with a statistically significant difference (HR = 0.43; 95% CI: 0.38–0.49; *P* < .001). Subgroup analyses based on whether the HR was adjusted for multiple factors yielded similar results (Figure S2, Supplemental Digital Content 6, http://links.lww.com/MD/M404). Subgroup analyses for different TACE types also showed consistent results across all subgroups.

#### 3.4.3. Adverse events

The meta-analysis results for treatment-related adverse events (TRAEs) of any grade are presented in Table 3. TRAEs mainly encompassed fever, abdominal pain, elevated ALT, elevated AST, hypertension, hand-foot syndrome, proteinuria, and diarrhea. None of the 8 indicators showed statistically significant results (*P* > .05). Significant heterogeneity was observed for abdominal pain (I^2^ = 76.9%, *P* < .001), while the statistical heterogeneity for the remaining indicators was minimal (I^2^ < 20.0%, *P* > .05). In the subgroup analysis for proteinuria, apart from the cTACE subgroup (RR = 1.49; 95% CI: 1.01–2.21; *P* = .044), the results for the other indicators were consistent with the overall pooled estimate.

**Table 3:** The meta-analysis results for treatment-related adverse events (TRAEs) of any grade.

TRAEs (any grade)	Studies	Heterogeneity *P* value I^2^ (%)		Meta-analysis RR (95% CI) *P* value		cTACE or DEB-TACE (RR/*P* value)	Subgroup results DEB-TACE (RR/*P* value)	cTACE (RR/*P* value)
Fever	10	0.694	0	0.99 (0.90,1.09)	0.895	0.95 (0.83,1.08) 0.405	1.10 (0.81,1.48) 0.54	1.04 (0.89,1.21) 0.649
Abdominal pain	9	0.000	76.9	1.29 (0.80,2.08)	0.305	3.29 (0.39,27.96) 0.275	1.02 (0.61,1.71) 0.929	0.94 (0.75,1.18) 0.618
Elevated ALT	7	0.899	0	1.19 (0.94,1.50)	0.151	0.87 (0.37,2.02) 0.742	1.18 (0.82,1.70) 0.364	1.25 (0.90,1.75) 0.185
Elevated AST	6	0.943	0	1.21 (0.94,1.56)	0.137	1.34 (0.60,2.98) 0.472	1.06 (0.73,1.53) 0.757	1.36 (0.93,2.00) 0.112
Hypertension	13	0.810	0	1.04 (0.92,1.17)	0.532	0.97 (0.84,1.12) 0.664	0.97 (0.44,2.12) 0.93	1.24 (0.99,1.56) 0.065
Hand-foot syndrome	11	0.944	0	0.93 (0.82,1.05)	0.227	0.89 (0.77,1.03) 0.131	0.70 (0.07,7.22) 0.761	1.10 (0.81,1.27) 0.901
Proteinuria	10	0.295	16.1	1.21 (0.91,1.61)	0.187	1.13 (0.66,1.93) 0.658	NA	1.44 (1.01,2.21) 0.044
Diarrhea	11	0.842	0	1.13 (0.92,1.37)	0.237	1.10 (0.88,1.38) 0.384	1.69 (0.52,5.43) 0.38	1.15 (0.73,1.80) 0.544

cTACE = conventional TACE, DEB-TACE = drug-eluting beads TACE, NA = not available, RR = relative risk.

The meta-analysis results for treatment-related adverse events (TRAEs) of grade 3 or above are presented in Table 4. None of the 8 indicators showed statistically significant results (*P* > .05), and there was no significant statistical heterogeneity (I^2^ = 0.0%, *P* > .05). In the subgroup analysis for hypertension, except for the DEB-TACE subgroup (RR = 4.72; 95% CI: 1.01–2.21; *P* = .018), the results of the other indicators were consistent with the overall pooled estimate.

**Table 4:** The meta-analysis results for treatment-related adverse events (TRAEs) of grade 3-5

TRAEs (≥3 grade)	Studies	Heterogeneity *P* value I^2^ (%)		Meta-analysis RR (95%CI) *P* value		cTACE or DEB-TACE (RR/*P* value)	Subgroup results DEB-TACE (RR/*P* value)	cTACE (RR/*P* value)
Fever	7	0.571	0	1.01 (0.49,2.08)	0.979	NA	0.69 (0.21,2.25) 0.539	1.01 (0.49,2.08) 0.612
Abdominal pain	6	0.978	0	0.93 (0.43,2.01)	0.857	1.03 (0.22,4.87) 0.971	0.70 (0.05,10.57) 0.797	0.93 (0.36,2.37) 0.879
Elevated ALT	8	0.995	0	1.36 (0.84,2.20)	0.209	2.93 (0.12,69.83) 0.507	1.31 (0.74,2.31) 0.355	1.42 (0.55,3.67) 0.464
Elevated AST	6	0.771	0	1.36 (0.80,2.32)	0.254	0.33 (0.01,7.76) 0.488	1.30 (0.70,2.41) 0.413	2.32 (0.54,9.88) 0.256
Hypertension	11	0.542	0	1.01 (0.75,1.37)	0.926	0.89 (0.63,1.26) 0.51	4.72 (1.31,16.99) 0.018	1.08 (0.56,2.07) 0.824
Hand-foot syndrome	7	0.906	0	0.96 (0.67,1.39)	0.845	0.87 (0.58,1.33) 0.529	NA	1.32 (0.62,2.79) 0.467
Proteinuria	5	0.803	0	1.00 (0.52,1.93)	0.998	0.92 (0.43,1.95) 0.826	NA	1.33 (0.34,5.22) 0.685
Diarrhea	6	0.723	0	0.96 (0.45,2.08)	0.927	0.59 (0.14,2.47) 0.472	1.64 (0.48,5.67) 0.433	0.80 (0.21,3.05) 0.74

cTACE = conventional TACE, DEB-TACE = drug-eluting beads TACE, NA = not available, RR = relative risk.

### 3.5. Sensitivity analysis

Sensitivity analysis was performed by sequentially excluding individual studies for the aforementioned outcome measures. The results illustrated that, following the exclusion of the study by Duan et al,^[23]^ the pooled results for proteinuria became statistically significant (RR = 1.53; 95% CI: 1.09–2.15; *P *< .05) (Figure S3, Supplemental Digital Content 7, http://links.lww.com/MD/M405). For the remaining outcome measures, excluding any single study resulted in consistent pooled results, indicating good stability of meta-analysis results.

### 3.6. Publication bias assessment

**Figure 8.**
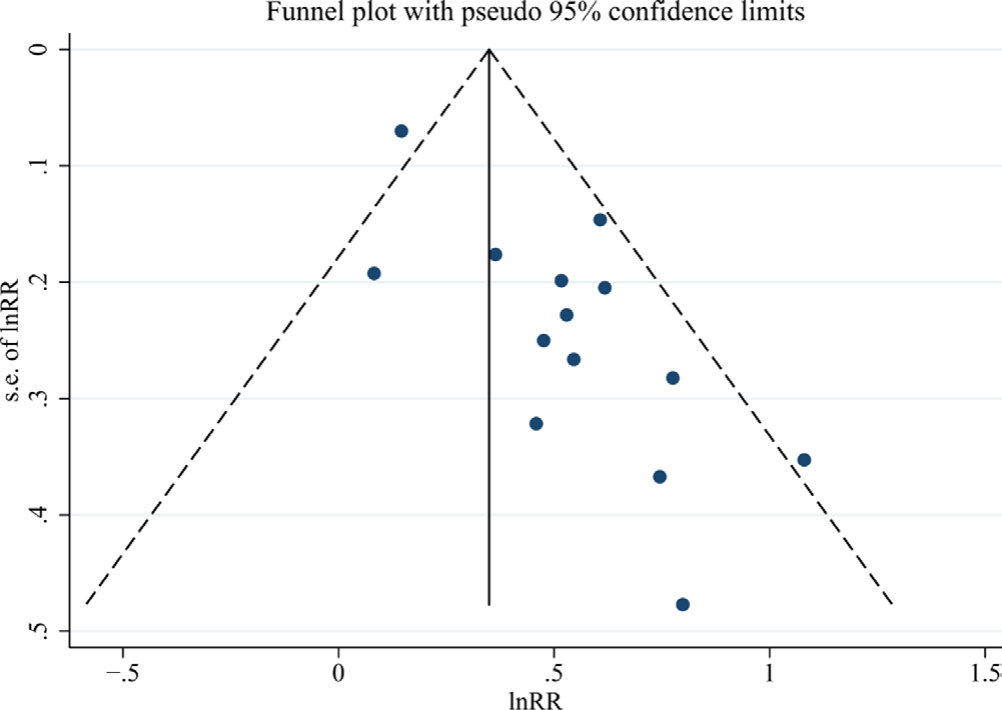
The funnel plot of objective response rate (ORR).

**Figure 9.**
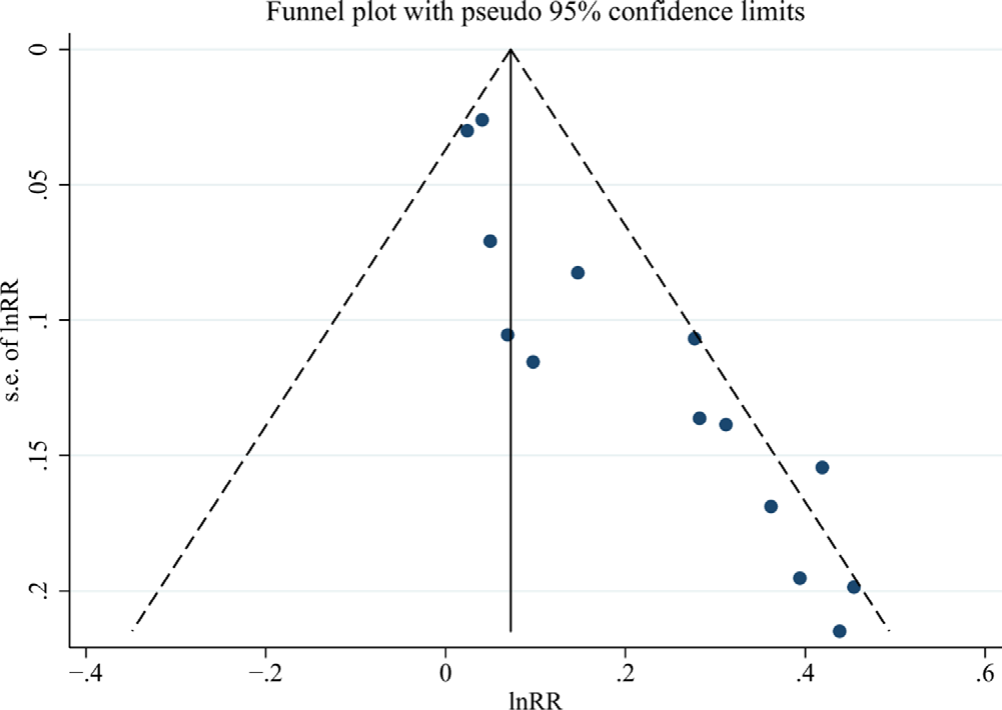
The funnel plot of disease control rate (DCR).

The efficacy indicators (ORR, DCR, PFS, OS) were the main observational outcome measures in this meta-analysis with a substantial number of included studies, and the funnel plots for all 4 indicators were asymmetrical, indicating the potential presence of publication bias. Egger test suggested significant publication bias for ORR and DCR (*P* < .05). Therefore, a trim-and-fill method was employed to assess the impact of publication bias on the pooled results. After imputing virtual studies, there was no reversal in the pooled results for the 2 indicators, indicating the stability of the results. The funnel plots of ORR and DCR are shown in Figures 8 and 9.

## 4. Discussion

Recently, significant breakthroughs have been achieved in the systemic treatment of advanced HCC, particularly with the notable efficacy of molecular-targeted therapy combined with immunotherapy.^[5,32–34]^ Local interventional therapies, represented by TACE, in conjunction with targeted therapy and immunotherapy, are emerging as new research hotspots and treatment approaches for intermediate and advanced HCC.^[35]^ Several studies have explored the differences in efficacy and safety between TACE-T-I and TACE-T, but the results have been inconsistent. This study, employing a meta-analysis approach, aims to assess the differences in efficacy and safety between these 2 treatment approaches, providing a more reliable basis for the treatment selection for unresectable HCC.

Thirteen retrospective cohort studies and one prospective real-world study were included in this study, involving a total of 2144 patients. The results suggested that compared to TACE-T, TACE-T-I demonstrated superior outcomes in terms of ORR, DCR, PFS, and OS. Among these outcome measures, OS was selected as the primary outcome in this Meta-analysis. And the results demonstrated a significant positive effect of this combination treatment (TACE-T-I) on patients’ overall survival. In addition, common treatment-related adverse events include fever, pain, abdominal pain, nausea, vomiting, elevated ALT, elevated AST, hypertension, hand-foot syndrome, proteinuria, and diarrhea. However, most adverse events did not show statistically significant differences between the 2 treatment approaches.

Prior studies have evaluated the clinical efficacy and survival benefits of TACE-T-I versus TACE-T for unresectable HCC. Regarding tumor response, the meta-analysis results of this study align with several studies,^[14,15,27,28,31]^ indicating that patients receiving triple therapy (TACE-T-I) exhibit higher ORR and DCR compared to those receiving dual therapy (TACE-T). However, Zhu et al^[18]^ found no significant differences in ORR and DCR between patients who received TACE-apatinib combination (TACE + A) and TACE-apatinib-camrelizumab combination (TACE + AC) (ORR: 55.9% vs 51.5%; DCR: 79.4% vs 72.1%; *P* > .05). Possible reasons include: The interval of TACE treatments may impact the outcomes; Some newly enrolled patients in the TACE + AC group received fewer cycles of PD-1 inhibitors; Patients with combined portal vein tumor thrombus are prone to extrahepatic metastasis, potentially diminishing the efficacy of immunotherapy. The study by Sun et al^[17]^ demonstrated that patients receiving triple therapy had a higher ORR than dual therapy (71% vs 42.3%; *P* = .023), although there was no significant difference in DCR between the 2 groups (93.5% vs 80.8%; *P* = .195). Also, the study by Jiang et al^[16]^ confirmed better tumor response of patients in the TACE-T-I group than TACE-T group, with a higher ORR (52.4% vs 17.8%; *P* = .001), but no significant difference in DCR (83.3% vs 77.8%; *P* = .514). This suggests that dual therapy may have an effect on stabilizing the tumor, and adding PD-1 inhibitors on this basis achieves tumor regression. In terms of survival benefits, the meta-analysis results of this study showed that compared to the TACE-T, TACE-T-I significantly prolongs the PFS and OS of patients with unresectable HCC, consistent with the results of the included studies.^[14–18,23,24,26–31]^ Subgroup analysis based on different TACE types aligns with the overall pooled results, suggesting that triple treatment regimen may offer better survival benefits for patients with unresectable HCC, unaffected by TACE type. The extension of OS in the TACE-T-I group may be related to the increased ORR, DCR, and prolonged PFS. Mechanistically, the potential reasons for the superior survival benefits observed in the TACE-T-I group are as follows: Synergistic effects of comprehensive treatment: TACE induces extensive local tumor necrosis, subsequently triggering an enhanced anti-tumor immune response through PD-1 inhibitors.^[36]^ The anti-proliferative and anti-angiogenic effects of multi-kinase inhibitors can counteract post-TACE neovascularization caused by hypoxia.^[37]^ Furthermore, they can modulate the tumor immune microenvironment, enhancing the immune response of PD-1 inhibitors in HCC.^[38,39]^ This leads to synergistic anti-tumor activity, improving the clinical prognosis of HCC patients. Immunostimulatory effects: Triple therapy can alleviate tumor burden, increase CD8^+^T cell infiltration, and alleviate the inhibitory effects of CD8^+^T cells, thereby initiating local and systemic immune activation, resulting in better survival benefits for patients with HCC.^[17]^

This study displayed no significant differences in treatment-related adverse events between the 2 groups, consistent with the results of multiple included studies. This suggests that the TACE-T-I regimen is safe and tolerable. However, the study by Chen et al^[15]^ demonstrated that among patients with grade ≥ 3 adverse events, the incidence of hypertension, nausea, and rash was higher in the TACE-T-I group than in the TACE-T group (*P* < .05). Additionally, in the subgroup analysis of some adverse events in this study, the TACE-T-I group showed a significantly higher incidence rate than the TACE-T group, with statistically significant differences, such as the cTACE subgroup for any grade proteinuria (RR = 1.49; 95%CI: 1.01–2.21; *P* = .044), and the DEB-TACE subgroup for grade 3 or above hypertension (RR = 4.72; 95%CI: 1.31–16.99; *P* = .018). This suggests that adding immunotherapy may increase the incidence of treatment-related adverse events. Therefore, in clinical practice, when choosing a treatment plan, consideration should be given to the potential increase in adverse events associated with adding immunotherapy.

The methodological quality of systematic reviews included in this research was moderate, with a low risk of attrition bias and reporting bias. Additionally, the differences in study participants and intervention protocols among the included studies were minimal, contributing to good generality of the meta-analysis results. However, several limitations exist in this study. First, some outcome indicators showed significant heterogeneity, and quantitative analysis did not identify influencing factors. Second, the studies included were all conducted in China, and the results may not necessarily apply to HCC patients in other Asian countries, Europe, or Africa. Third, due to limitations in the number of included studies and sample size, no significant differences were found in the incidence of adverse reactions. Fourth, most included studies were retrospective cohort studies, with only one prospective real-world study. Therefore, further high-quality large RCTs are needed in the future to further validate the analysis results.

Collectively, TACE-T-I may offer superior clinical efficacy and survival benefits for patients with unresectable HCC, and the regimen appears to be well-tolerated. This provides a theoretical basis for clinical treatment decisions for intermediate and advanced HCC. Meanwhile, additional high-quality research is required to verify this finding. In addition, further stratification of patients with intermediate and advanced HCC is required to identify those who would benefit from the triple combination therapy.^[40]^

## Author contributions

**Data curation:** Jingwen Feng, Yi Zhao.

**Formal analysis:** Jingwen Feng.

**Project administration:** Lin Zhai, Jingxu Zhou.

**Software:** Jingwen Feng.

**Writing – original draft:** Jingwen Feng.

**Writing – review & editing:** Jingxu Zhou.

## Supplementary Material









**Figure:**
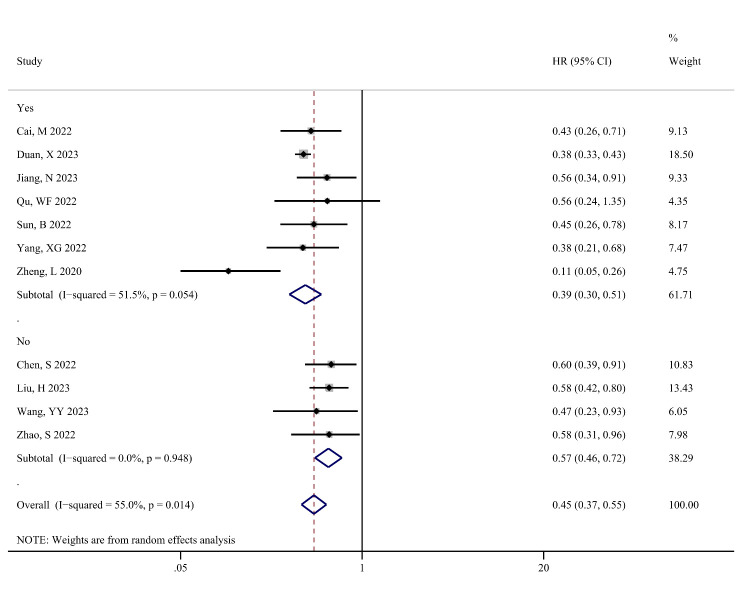


**Figure:**
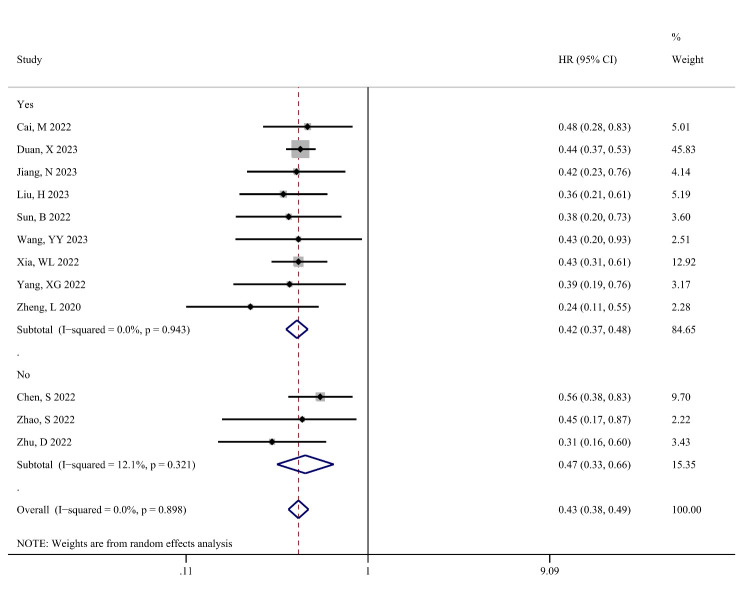


**Figure:**
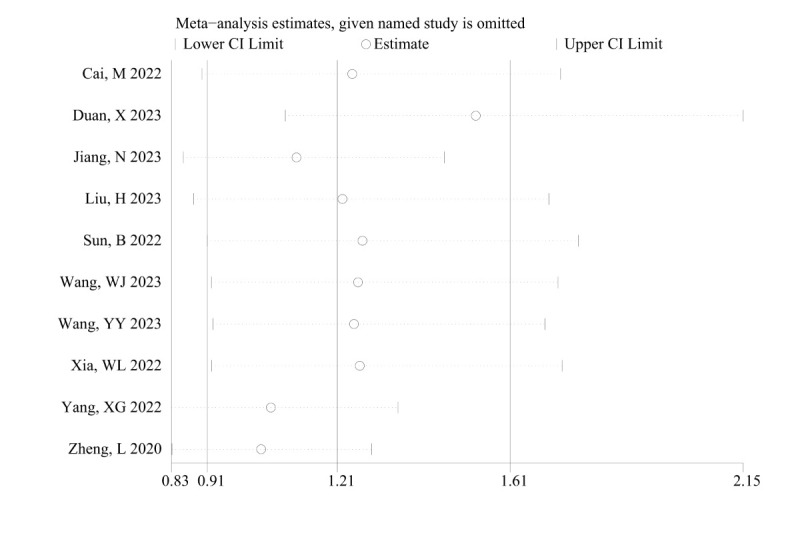

