# Supplementary material for: Efficacy and safety of transarterial chemoembolization combined with targeted therapy and immunotherapy versus with targeted monotherapy in unresectable hepatocellular carcinoma: A systematic review and meta-analysis
Source: Medicine (Baltimore). 2024 May 3;103(18):e38037. doi: 10.1097/MD.0000000000038037 (PMC11062670; doi:10.1097/MD.0000000000038037)
Supplement: Supplementary file 1 [file medi-103-e38037-s001.docx]

**Search Strategy**

**Supplementary Table 1 PubMed**

| Search | Query | Items found |
| --- | --- | --- |
| #1 | ("liver neoplasms"[MeSH Terms] OR "liver neoplasms"[All Fields] OR "liver cancer"[All Fields] OR "carcinoma, hepatocellular"[MeSH Terms] OR "hepatocellular carcinoma"[All Fields]) | 241499 |
| #2 | ("transcatheter arterial chemoembolization"[All Fields] OR "tace"[All Fields] OR "transhepatic arterial chemoembolization"[All Fields] OR "transarterial chemoembolization"[All Fields]) | 9760 |
| #3 | ("target"[All Fields] OR "targeted"[All Fields] OR "sorafenib"[Supplementary Concept] OR "sorafenib"[All Fields] OR "sorafenib"[MeSH Terms] OR "lenvatinib"[Supplementary Concept] OR "lenvatinib"[All Fields] OR "regorafenib"[Supplementary Concept] OR "regorafenib"[All Fields] OR "apatinib"[Supplementary Concept] OR "apatinib"[All Fields] OR "bevacizumab"[Supplementary Concept] OR "bevacizumab"[All Fields] OR "bevacizumab"[MeSH Terms]) | 1534322 |
| #4 | ("immunotherapy"[MeSH Terms] OR "immunotherapy"[All Fields] OR "immunotherapies"[All Fields] OR "immunological therapy"[All Fields] OR "immune checkpoint inhibitors"[Pharmacological Action] OR "immune checkpoint inhibitors"[MeSH Terms] OR "immune checkpoint inhibitors"[All Fields]) OR "pd 1 inhibitor"[All Fields] OR "pd l1 inhibitor"[All Fields] OR ("atezolizumab"[Supplementary Concept] OR "atezolizumab"[All Fields] OR "pembrolizumab"[Supplementary Concept] OR "pembrolizumab"[All Fields] OR "nivolumab"[Supplementary Concept] OR "nivolumab"[All Fields] OR "nivolumab"[MeSH Terms] OR "camrelizumab"[Supplementary Concept] OR "camrelizumab"[All Fields] OR "sintilimab"[Supplementary Concept] OR "sintilimab"[All Fields] OR "toripalimab"[Supplementary Concept] OR "toripalimab"[All Fields]) | 444236 |
| #5 | #1 AND #2 AND #3 AND #4 | 240 |
