# Supplementary material for: Efficacy and safety of transarterial chemoembolization combined with targeted therapy and immunotherapy versus with targeted monotherapy in unresectable hepatocellular carcinoma: A systematic review and meta-analysis
Source: Medicine (Baltimore). 2024 May 3;103(18):e38037. doi: 10.1097/MD.0000000000038037 (PMC11062670; doi:10.1097/MD.0000000000038037)
Supplement: Supplementary file 2 [file medi-103-e38037-s002.docx]

**Supplementary Table 2 Embase**

| Search | Query | Items found |
| --- | --- | --- |
| #1 | ('liver neoplasms'/exp OR 'liver neoplasms':ti,ab OR 'liver neoplasm'/exp OR 'liver neoplasm':ti,ab OR 'liver cancer'/exp OR 'liver cancer':ti,ab OR 'hepatocellular carcinoma'/exp OR 'hepatocellular carcinoma':ti,ab) | 338391 |
| #2 | ('transcatheter arterial chemoembolization'/exp OR 'transcatheter arterial chemoembolization' OR tace:ti,ab OR 'transhepatic arterial chemoembolization' OR 'transarterial chemoembolization'/exp OR 'transarterial chemoembolization') | 25410 |
| #3 | (targeted:ti,ab OR 'sorafenib'/exp OR sorafenib OR 'lenvatinib'/exp OR lenvatinib OR 'regorafenib'/exp OR regorafenib OR 'apatinib'/exp OR apatinib OR 'bevacizumab'/exp OR bevacizumab) | 647731 |
| #4 | ('immunotherapy'/exp OR immunotherapy:ti,ab OR immunotherapies:ti,ab OR 'immunological therapy'/exp OR 'immunological therapy':ti,ab OR 'immune checkpoint inhibitors'/exp OR 'immune checkpoint inhibitors':ti,ab OR ('pd 1 inhibitor'/exp OR 'pd 1 inhibitor' OR 'pd l1 inhibitor'/exp OR 'pd l1 inhibitor') OR 'atezolizumab'/exp OR atezolizumab OR 'pembrolizumab'/exp OR pembrolizumab OR 'nivolumab'/exp OR nivolumab OR 'camrelizumab'/exp OR camrelizumab OR 'sintilimab'/exp OR sintilimab OR 'toripalimab'/exp OR toripalimab) | 376306 |
| #5 | #1 AND #2 AND #3 AND #4 | 1099 |
