# Supplementary material for: Efficacy and safety of transarterial chemoembolization combined with targeted therapy and immunotherapy versus with targeted monotherapy in unresectable hepatocellular carcinoma: A systematic review and meta-analysis
Source: Medicine (Baltimore). 2024 May 3;103(18):e38037. doi: 10.1097/MD.0000000000038037 (PMC11062670; doi:10.1097/MD.0000000000038037)
Supplement: Supplementary file 3 [file medi-103-e38037-s003.docx]

**Supplementary Table 3 Web of Science**

| Search | Query | Items found |
| --- | --- | --- |
| #1 | "Liver Neoplasms" OR "Liver Neoplasm" OR "liver cancer" OR "hepatocellular carcinoma" (All Fields) | 169763 |
| #2 | "transcatheter arterial chemoembolization" OR TACE OR "transhepatic arterial chemoembolization" OR "transarterial chemoembolization" (All Fields) | 11915 |
| #3 | Targeted OR sorafenib OR Lenvatinib OR regorafenib OR apatinib OR bevacizumab (All Fields) | 2465300 |
| #4 | immunotherapy OR immunotherapies OR "immunological therapy" OR "immune checkpoint inhibitors" OR "pd 1 inhibitor" OR "pd l1 inhibitor" OR Atezolizumab OR pembrolizumab OR nivolumab OR camrelizumab OR sintilimab OR toripalimab (All Fields) | 205200 |
| #5 | #1 AND #2 AND #3 AND #4 | 394 |
