# Supplementary material for: Efficacy and safety of transarterial chemoembolization combined with targeted therapy and immunotherapy versus with targeted monotherapy in unresectable hepatocellular carcinoma: A systematic review and meta-analysis
Source: Medicine (Baltimore). 2024 May 3;103(18):e38037. doi: 10.1097/MD.0000000000038037 (PMC11062670; doi:10.1097/MD.0000000000038037)
Supplement: Supplementary file 4 [file medi-103-e38037-s004.docx]

**Supplementary Table 4 The Cochrane library**

| Search | Query | Items found |
| --- | --- | --- |
| #1 | MeSH descriptor: [Liver Neoplasms] explode all trees | 3920 |
| #2 | MeSH descriptor: [Carcinoma, Hepatocellular] explode all trees | 2399 |
| #3 | (Liver Neoplasms OR Liver Neoplasm OR liver cancer OR hepatocellular carcinoma) :ti,ab,kw (Word variations have been searched) | 17719 |
| #4 | #1 OR #2 OR #3 | 17719 |
| #5 | ("transcatheter arterial chemoembolization" OR TACE OR "transhepatic arterial chemoembolization" OR "transarterial chemoembolization"): ti,ab,kw (Word variations have been searched) | 1571 |
| #6 | MeSH descriptor: [Sorafenib] explode all trees | 632 |
| #7 | (Targeted OR sorafenib OR Lenvatinib OR regorafenib OR apatinib OR bevacizumab): ti,ab,kw (Word variations have been searched) | 119183 |
| #8 | MeSH descriptor: [Bevacizumab] explode all trees | 2650 |
| #9 | #6 OR #7 OR #8 | 119183 |
| #10 | MeSH descriptor: [Immunotherapy] explode all trees | 11744 |
| #11 | MeSH descriptor: [Immune Checkpoint Inhibitors] explode all trees | 196 |
| #12 | (immunotherapy OR immunotherapies OR "immunological therapy" OR "immune checkpoint inhibitors" OR "pd 1 inhibitor" OR "pd l1 inhibitor" OR Atezolizumab OR pembrolizumab OR nivolumab OR camrelizumab OR sintilimab OR toripalimab): ti,ab,kw (Word variations have been searched) | 19947 |
| #13 | #9 OR #10 OR #11 | 27737 |
| #14 | #4 AND #5 AND #9 AND #13 | 72 |
| #15 | #14 in Trials | 72 |
